# Assessing the impact of COVID-19 on access to family planning and HIV services among women in Lilongwe, Malawi

**DOI:** 10.1371/journal.pgph.0007041

**Published:** 2026-08-11

**Authors:** Clara Lemani, Agatha Bula, Annie Thom, Jennifer H. Tang

**Affiliations:** 1 UNC Project-Malawi, Lilongwe, Malawi; 2 School of Medicine, University of North Carolina at Chapel Hill, Chapel Hill, North Carolina, United States of America; Universidad Nacional de Colombia, COLOMBIA

## Abstract

Following its declaration as a pandemic, COVID-19 spread extensively, affecting millions globally. Concerns emerged that it could have a detrimental impact on sexual and reproductive health services. Specifically, these worries centred on the potential disruption of family planning and HIV-related care. We conducted a mixed methods study to understand the impact of the pandemic and its associated measures on access to FP and HIV services among women in a peri-urban area of Lilongwe, Malawi. We used descriptive statistics to assess what proportion of eligible women accessed FP/HIV services and understand experiences and challenges faced. 400 women responded to a questionnaire, and 30 of them completed in-depth interviews. We used frameworks by Peters (2008) and; Ensor and Cooper (2004) to analyse qualitative data. The mean age of the study participants was 30 years, with less than a third aged 18–25. Of the 269 who tried to access FP services, 254 (94.4%) received it, and 248 (92.2%) received their preferred method. Only 2 (6.1%) out of 33 women living with HIV reported difficulties in obtaining ART. For HIV Testing & Counselling (HTC) services, 97.0% (n = 293) of the 302 women who wanted the service attempted to access them, and 99.0% (n = 290) of the 293 were successful. The most commonly reported challenges were: being sent away if they did not have a mask, being sent away if the maximum number of clients per day had been reached, and higher fares for the few who used minibus. Overall, access to FP and HIV services was sustained. Women, however, faced some challenges while accessing the services, which were more pronounced among FP clients compared to ART clients. Our findings emphasize the need to implement strategies that will minimize challenges to health care access during pandemics, to ensure gains in FP and HIV services are sustained.

## Background

Following its declaration as a pandemic by the World Health Organization (WHO) in March 2020, COVID-19 spread extensively, affecting millions globally. Health systems were challenged, and resources and staff were diverted to the fight against the pandemic. Measures put in place to reduce further spread of the virus created both logistical and economic barriers to accessing health services. Fear of infection, lockdowns, travel restrictions, and economic hardships due to the virus prevented people from accessing health services. There were concerns that the impact of COVID-19 on Sexual and Reproductive Health Services (SRHS), specifically Family Planning (FP) and Human Immunodeficiency Virus (HIV) services, could be detrimental [1–3].

The United Nations Population Fund (UNFPA) estimated that about 12 million women globally may have been unable to access FP services because of the pandemic, leading to 1.4 million unintended pregnancies [4]. While some African countries maintained consistent family planning services through innovative adaptations, others experienced significant service interruptions due to lockdowns and mobility restrictions [5,6]. Women reported to have had challenges accessing FP during the pandemic [7,8], with some citing closure of the clinic as their main reason [8]. Travel restrictions prevented mobile outreach teams to provide services to rural communities and the ability of clients to access services [9,10]. These disruptions led to a disproportionate impact on access to FP, with the rural areas more likely to suffer [10].

The COVID-19 pandemic introduced changes in fertility desires across Africa, driven more by socioeconomic instability than direct health fears. While global trends often pointed toward a decline in birth intentions, research in Sub-Saharan Africa suggests that fertility preferences remained stable at the population level during the first year of the crisis [11]. However, significant individual-level shifts occurred; for instance, in Burkina Faso and Kenya, there was a documented increase in contraceptive use as women sought to navigate the uncertainties of the pandemic [5]. These changes were heavily influenced by economic factors, as evidenced in Nigeria, where women facing severe food insecurity were nearly three times more likely to change their pregnancy plans [12]. Furthermore, while some women opted to delay childbearing due to income loss, others with chronic food insecurity in Kenya surprisingly showed higher odds of wanting to accelerate their next birth, possibly viewing children as a form of future social security [13]. Ultimately, the pandemic acted as a catalyst for increased demand for family planning services as women sought greater control over the timing and spacing of births during a period of intense financial strain [14].

In addition, COVID-19 also affected access to HIV services, with some countries reporting moderate to very high levels of disruption [15]. In most published studies to date, a decline in HIV testing, HIV prevention services, and all other aspects of treatment and care were observed [2,16–20]. For example, a South African study of 65 primary care clinics reported a 47.6% decline in HIV testing, while a 54.7% decline in HIV testing was observed at a clinic in Guatemala [16,17]. Some studies also reported antiretroviral therapy (ART) stock outs due to logistical challenges [20].

Disruptions to HIV services lasting over 6 months could cause a 10% increase in deaths in high-burden nations over 5 years, largely due to antiretroviral therapy (ART) interruptions [1,21]. Specifically, a 6-month ART supply failure affecting half of the treated population is estimated to increase HIV-related deaths by 1.63 times [2,3]. A compilation of experiences of people living with HIV during the COVID-19 pandemic by WHO and UNAIDS highlighted key challenges, which included lack of information about the pandemic, lack of clarity on measures to ensure continuity of services, low stock of medicine and decreased accessibility of HIV services [20].

Malawi is a land-locked country in Sub-Saharan Africa with an HIV prevalence of 10% among women aged 15–49 years [22] In Malawi, COVID-19 was first reported on 2 April 2020, and there was ultimately a total of 85,452 confirmed cases and 2,619 related deaths [23]. The Malawi government announced measures aimed at reducing the spread of COVID-19 before the first case was confirmed, which included banning gatherings of more than 100 people, closure of schools, enforcing social distancing and wearing of masks and self-quarantine for people coming from affected areas. All these measures were implemented countrywide, including at health facilities.

The Malawi Ministry of Health’s (MoH) Department of HIV & AIDS issued multiple guidance documents on provision of HIV services during the COVID-19 pandemic. Its first guidance (issued 3 April 2020) recommended suspension of non-essential services, such as routine scheduled viral load monitoring for uncomplicated stable patients, patient support groups that involved gathering of people, and active tracing involving community visits. It also recommended additional measures, which included opening clinics each day of the week and 6-month dispensing of ART for as many patients as possible [24]. Its second guidance (issued 17 April 2020) also suspended routine voluntary counselling and testing (VCT), active partner notification, condom distribution to walk-in clients and community HIV testing services [25]. All of these services were suspended until 14 August 2020, after which they were only allowed to resume if adequate personal protective equipment was available [26].

Given the disruptions to FP and HIV care in Malawi during the COVID-19 pandemic, we conducted this mixed methods study to understand the impact of the pandemic and its associated measures on access to FP and HIV services among women living in Area 25 of Lilongwe. Specifically, we sought to assess: (1) The impact of COVID-19 on access to ART; (2) The impact of COVID-19 on access to HIV Testing & Counselling (HTC) services; (3) The effect of COVID-19 on FP access; (4) The proportion of unintended pregnancies, and (5) Barriers to accessing HIV and FP-related services.

## Methods

### Study design

We employed both quantitative and qualitative methods to assess what proportion of eligible women accessed FP/HIV services and understand their motivations, experiences, and challenges while accessing or trying to access the services. Household surveys were used to collect quantitative data, and a subset of these respondents were invited to also complete in-depth interviews (IDIs) for qualitative data collection. Survey questions to capture demographic details, HIV status, and the utilization of family planning (FP) and HIV services during the COVID-19 pandemic were created by the study team and based on other validated household surveys conducted in Malawi, such as the Demographic and Health Surveys. Questions were adapted to ask about participant experiences while accessing or trying to access services during the COVID-19 pandemic. We piloted the tools with a small group before main study data collection to ensure they were clear. Data collection was conducted between 2 October 2023 and 9 January 2024.

### Conceptual framework

**Fig 1:**
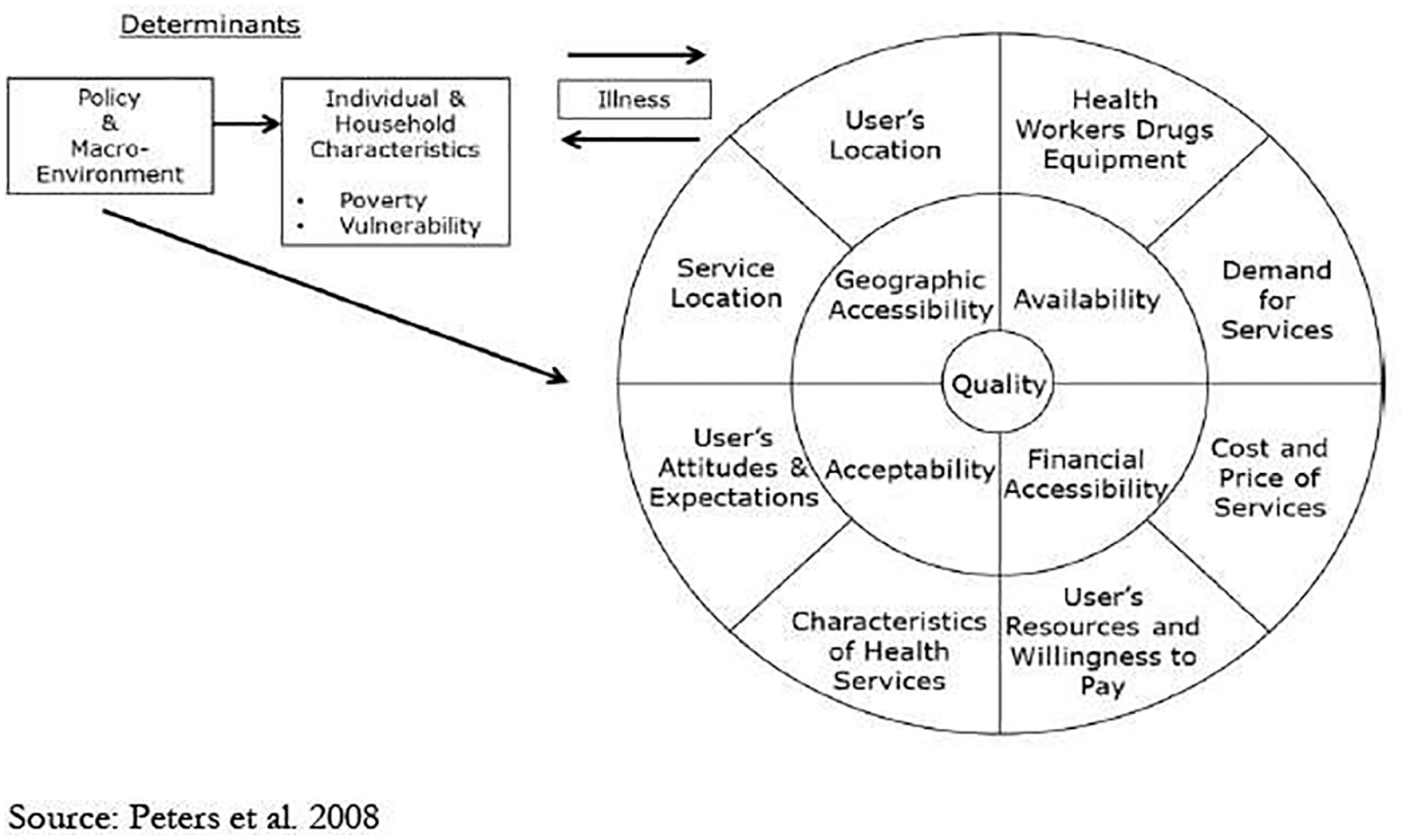
Conceptual framework for assessing barriers to accessing health services.

We adapted a framework developeSess barriers to accessing health services within implementation strategies [27] (Fig 1), as well as a framework by Ensor and Cooper (2004) that focuses on demand side barriers to health services access [28]. The two frameworks were adapted to suit our context and research questions.

### Study setting and population

The participants were recruited from Area 25, a residential peri-urban area north of Lilongwe city. It is a high-density area with an estimated population of 107,316 [29]. The area has a mixed population consisting of civil servants, non-governmental organization employees, and casual and informal sector workers [30]. The area is serviced by a government community hospital, a mission hospital, and other small private clinics.

The study population was comprised of those who: 1) Were willing to participate in and consent for the study; 2) Were aged 18–45 years; 3) Had been residing in Area 25 at least for the past 2 years; 4) Were willing to take medications (they were not prohibited by religious or personal beliefs); and 5) Could communicate in Chichewa (the most common local language) or English. The study was approved by the UNC Institutional Review Board on 22 August 2022 (Reference number: 349880) and the Malawi National Health Science Research Committee on 18 January 2023 (Reference number: 2901). Informed written consent was obtained from all individual participants included in the study. All participants were asked to sign a written informed consent form prior to data collection. To ensure full understanding, the interviewers read and explained the consent form to each participant and addressed any questions they had regarding the study’s purpose and procedures. In cases where participants were illiterate, informed consent was obtained in the presence of an impartial witness. For these participants, agreement was documented via a participant’s thumbprint and then signed and dated by the witness to indicate that the information was accurately explained and the participant understood. Participants received 2,500 Malawi Kwacha as a compensation for their time in the study, and those who participated in IDI also received a drink and snack as the IDI took more time to complete.

### Sampling and sample size

For the household survey, three trained interviewers administered questionnaires to the oldest woman (usually the primary caretaker) found in the household, if she consented. Households were selected using systematic sampling, in which every 10^th^ household (where 10 was the sampling interval) was interviewed [31]. The first household was identified randomly from a central point. Selection then continued in every 10^th^ household in all directions. If a respondent was not available or did not consent, the interviewer continued to the next 10^th^ household. Our target population was estimated at 30370 women based on the fact that 28.3% of the urban population is comprised of women aged 15–49 [29] A total of 400 women responded to the quantitative survey. The sample size was calculated using simple random sampling using a 95% confidence level, 5% precision, and the assumption of an unintended pregnancy prevalence of 41% [32]. We included the prevalence rate to ensure that the sample size was powered to estimate the outcome of unintended pregnancy within our population during the pandemic. While the initial sample size was powered based on the prevalence of unintended pregnancies, the resulting data yielded an HIV prevalence estimate (8.3%, 95% confidence interval [5.7%-11.4%]) that closely aligns with the known rates in the general population of 10% [22]. Because these prevalence figures are comparable, the current sample size is statistically robust enough to provide a reliable estimation of HIV service access alongside our primary unintended pregnancy findings.

A subset of 30 women who completed the surveys were purposively selected to take part in IDIs. These women were identified by the quantitative interviewers based on their willingness to take part in the IDIs, and our recruitment needs for interviewing women for our four study groups: (1) Those who accessed FP services, (2) Those who did not access FP Services, (3) Those who had access to HIV services (4) Those who did not access HIV services. Those who accessed FP and HIV services gave insight on their experiences as they accessed the services and whether those experiences were influenced by the pandemic and its restrictions. Those who did not access the services helped us understand whether they could not do so due to COVID-19 and its restrictions. Data collection continued until thematic saturation was achieved. Saturation was defined as the point at which no new themes or insights emerged from three consecutive interviews within each purposive group. Preliminary analysis of the 10 interviews per group confirmed that the breadth and depth of experiences regarding FP and HIV service usage during the pandemic had been sufficiently captured.

### Data collection and analysis

#### Quantitative data.

Quantitative data was collected in October 2023 using the ODK platform on a password-protected tablet. The forms were then sent to a UNC Project-Malawi server hosting the ODK Aggregate tool and then accessed as an XML file by study personnel. We used ODK to ensure data quality as the pre-coded skip patterns, ranges, and restrictions tailored to the survey’s needs reduce data collection errors [33]. To ensure data completeness, we minimized missing values by configuring all Open Data Kit (ODK) fields as mandatory, while incorporating ‘No Response’ or ‘Declined’ options to respect participant autonomy without leaving gaps in the dataset. Data were then exported into Stata version 18 (StataCorp LLC, College Station, Texas, USA) for analysis. We used descriptive statistics to report frequencies and unweighted proportion distributions of the study population stratified by each of the covariates in the univariate analysis. Unweighted proportions are acceptable in this case because we used simple random sampling, where every household had an equal chance of selection, making the data self-weighted. Additionally, the non-response rate was negligible, eliminating the need for weighting adjustments [34].

#### Qualitative data.

Qualitative data were collected from 21 November 2023–9 January 2024. A trained interviewer visited the identified households to confirm if they were still willing to participate in the IDI. Another consent was administered, and the participants chose whether to have the interview conducted in their house or another private area. A pre-developed interview guide was used. The guide included questions about concerns about COVID-19 and its impact on daily lives, as well as motivations and experiences while accessing or trying to access FP and/or HIV services. IDIs were conducted in Chichewa by experienced qualitative researchers from UNC Project-Malawi. All interviews were audio-recorded and lasted 20 minutes on average. All were transcribed and translated into English for analysis.

All the transcripts were verified by the study Principal Investigator, and any discrepancies were discussed with the interviewer. A codebook was developed iteratively and collaboratively by the team members using the interview guide and was further refined inductively through reading the transcripts. The codebook was generated inductively from the data to capture participants’ perspectives without imposing predefined categories. All transcripts were double-coded using NVivo software version 14.0 achieving intercoder agreement of 95%. Any coding discrepancies of below the 95% threshold were discussed and reconciled. Coded data were then thematically analysed to identify emergent themes, and analytical memos were written to summarize and interpret the findings. Once themes emerged, the conceptual framework was used to interpret, organize, and situate these findings within a broader theoretical lens. This approach allowed us to remain grounded in the data while also ensuring theoretical coherence. By mapping inductive codes back to the framework, we maintained rigor and demonstrated how participant experiences align with and extend existing theory.

## Results

### Quantitative results

The mean age of the 400 study participants was 30 years, with less than a third aged 18–25 years (Table 1). Most women were married and had 1–2 children. Most women and their partners had completed at least some secondary school or more. When asked about FP use, 356 (89.0%) women reported ever using FP, of which 275 (77.2%) were using FP at the time Malawi started recording COVID-19 cases (Table 2). Most (n = 269, 97.5%) of the 276 women who wanted FP tried to access it. Of the 269 who tried to access FP services, 254 (94.4%) received it, and 248 (92.2%) received their preferred method; 43 (16.0%) of the 269 women said that COVID-19 affected their choice of FP method. However, 51 (18.5%) of 276 women who wanted FP said that COVID-19 delayed or caused a cancellation of an FP visit, and 84 (30.4%) said it affected their choice of where they tried to access it. Almost half of the 400 women (n = 194, 48.5%) became pregnant during the pandemic; of these 194, 65 (33.5%) said that the pregnancy was unintended.

**Table 1:** Description of demographic and socio-economic characteristics.

Characteristic	Number (%)
Age	
18–25 years	132 (33.0)
26–35 years	170 (42.5)
36–45 years	98 (24.5)
Marital status	
Single	26 (6.5)
Married	315 (78.8)
Divorced	46 (11.5)
Widowed	13 (3.2)
Total children	
None	12 (3.0)
1–2 children	241 (60.3)
3–5 children	136 (34.0)
More than 5 children	11 (2.7)
Desire for more children	
None	150 (37.5)
1–2 children	191 (47.8)
3–5 children	57 (14.2)
More than 5 children	2 (2)
Participant education level	
Completed Primary or less	146 (36.5)
Some secondary	119 (28.8)
Completed Secondary or more	135 (33.7)
Partner’s education level	
Completed Primary or less	42 (13.3)
Some secondary	58 (18.4)
Completed Secondary or more	212 (67.3)
Don’t know	3 (1)
Participant occupation	
Farming	5 (1.3)
Salaried work	45 (11.2)
Business	158 (39.5)
Student	5 (1.3)
None	179 (44.7)
Other	8 (2.0)
Partner’s occupation	
Farming	3 (1)
Salaried work	189 (60.0)
Business	91 (28.9)
Student	1 (0.3)
None	16 (5.1
Other	15 (4.7)
Average monthly income	
Less than MK50,000	174 (43.5)
MK50,000- MK100,000	127 (31.7)
MK100,001- MK150,000	43 (10.8)
MK150,001 or more	56 (14.0)

**Table 2:** Family Planning and HIV Service Use and Related Outcomes among 400 Women in Lilongwe, Malawi during the COVID-19 pandemic.

Variable	Yes Number (%) [ci])	Total N
**Ever Use of FP**	356 (89.0 [85.5, 91.9])	400
Were using FP when COVID started (April 2020)	275 (77.2 [72.5, 81.5])	356
**Got pregnant during COVID-19 pandemic**	194 (48.5 [43.5, 53.5])	400
Pregnancy was unintended	65 (33.5 [26.9, 40.6])	194
**Wanted FP during COVID-19 pandemic**	276 (69.0 [64.2, 73.5])	400
Tried to access FP (out of those who wanted FP)	269 (97.6 [94.8, 99.0])	276
Cancelled/delayed FP visit due to COVID-19	51 (18.5 [14.1, 23.5])	276
COVID 19 affected choice of where they tried to access FP (source)	84 (30.4 [25.1, 36.2])	276
Obtained FP (out of those who tried)	254 (94.4 [91.0, 96.8])	269
COVID-19 affected choice of FP method	43 (16.0 [11.8, 20.9])	269
Received preferred method	248 (92.2 [88.3, 95.1])	269
COVID-19 affected why they did not receive preferred method	17 (81.0 [58.1, 94.6])	21
**Eligible for HTC during COVID-19 pandemic**		367
Wanted HTC during COVID-19 pandemic	302 (82.3 [78.0, 86.1])	367
Attempted to access HTC	293 (97.0 [94.4, 98.6])	302
Accessed HTC	290 (98.9 [97.0, 99.8])	293
COVID-19 restrictions affected access to HTC	95 (31.5 [26.4, 37.3])	302
**Eligible for ART during COVID-19 pandemic**		33
Attempted to access ART	33 (100)	33
Accessed ART	33(100)	33
Had difficulty obtaining ART	2 (6.1 [0.7, 20.2])	33
Diagnosed with ART resistance	2 (6.1 [0.7, 20.2])	33
COVID-19 restrictions affected ART access	2 (6.1 [0.7, 20.2])	33

FP = Family Planning; HTC = HIV Testing and Counselling; ART = Antiretroviral Therapy.

In terms of HTC services, 302 (82.3%) of the 367 women who were eligible for the service wanted to access them during the pandemic (Table 2). Of these 302, 293 (97.0%) attempted to access them, and 99.0% (n = 290) of the 293 were successful**.** Overall, 31.5% (n = 95) of the 302 women reported that COVID-19 restrictions affected their access to HTC services.

All women were asked about their HIV status; 33 reported being HIV positive, resulting in an HIV prevalence of 8.3% among the study population. All 33 of these women were on ART. Only 2 (6.1%) out of these 33 women living with HIV reported difficulties in obtaining ART and believed that they were due to the pandemic and its associated measures (Table 2). There were also 2 women who reported being diagnosed with ART resistance.

To check the precision of our primary estimates, we calculated standard errors (SE) and relative standard errors (RSE) and compared them with standard benchmarks for reproductive health surveys (S1 Table). All calculated RSEs for the Family Planning (FP) and HIV Testing and Counseling (HTC) indicators were below 20%. This indicates high precision with reference to the data quality benchmarks established by the National Center for Health Statistics [35], which state that an RSE under 30% denotes a statistically stable estimation. Conversely, the calculated RSE for the Antiretroviral Therapy (ART) estimate was high (68.5%). This high relative variance occurred because the sample size for this specific subgroup was very small (n = 33) and the proportion of positive cases was also small (n = 2), making the estimate highly unpredictable. These specific ART results should, therefore, be interpreted with extreme caution.

### Qualitative results

While our quantitative findings indicate that women continued to access FP and HIV services despite fears of COVID-19, the qualitative phase explores the underlying experiences of these clients and identifies the specific barriers faced by those who did not seek care. Qualitative analysis was performed guided by the themes and sub-themes derived by the two frameworks used (Table 3)

**Table 3:** Themes and subthemes for qualitative analysis.

Geographical Accessibility
• Household location • Distance to local FP and ART clinics
Availability
• Health worker, drug, and equipment supply at local clinics • Clinic opening and waiting times at local clinics for FP and ART • Information given about FP and ART options at local clinics • Quality of staff and service provision at local clinics
Acceptability
• User’s attitudes and expectations about FP and ART service provision • Household expectations for utilization of FP and ART services • Community and cultural beliefs and attitudes about FP and ART service provision • Most important characteristics for FP and ART health services
Financial Accessibility
• User’s resources and household employment during pandemic • User’s willingness and ability to pay for and access transport to clinics and health services or supplies versus need to prevent unintended pregnancy or HIV resistance

### Geographical location

The participants were asked how they got from their home to their clinics and what challenges they faced to get to the clinics during the COVID-19 pandemic. They were further asked if they were affected by the new limits for the number of people in a minibus, which were introduced to prevent the further spread of the virus.

Most of them reported that they did not face any challenges to get to their clinics because they lived within walking distances. Very few reported that the distance from their home required them to board a minibus.

*“That did not really affect me because the hospital I go to is Area 25 health center, and I go by foot. I left home, walked to the hospital, and that was not a problem…” (Participant 003)*

*“There were no challenges in terms of travelling. We would walk.” (Participant 023).*

### Availability of services

We asked about the availability of drugs/FP commodities and also about the availability of staff to offer the services. A majority of those seeking ART services reported that the service was available when they needed it. In contrast, only half of those that tried to access FP and HTC services reported that the services they were looking for were available on their first attempt:

*“I found the service I was looking for. I use the injection method and when I went, I found the method I wanted. They injected me and I went back home.” (Participant* 020)

The other half reported that the services were either completely unavailable, or they had to go to the clinic more than once to access them. This was mostly because the FP clinics had a specified number of clients they could assist in a day, to avoid overcrowding and further spread of the virus. Clients were turned away when a specific number for the day had been reached. Other women reported FP commodity stockouts when they tried to access their preferred method. Both barriers increased the risk of unintended pregnancies, as noted by these women:

*“We used to go, but, when we go there, we were not receiving the services. We would go to the hospital, but they would send us back saying ‘we have no things here because of COVID,’ we go the next month and they were still telling us the same thing* #.” *(Participant 001)*

*“I tried to get help at the hospital since we are close to one, the Area 25 clinic, but I could return home without being assisted until I got pregnant, but I did not plan to get pregnant, so I aborted.” (Participant 010)*

### Acceptability

#### User’s attitudes and expectations.

Most of the participants expected continuity of FP and HIV services during the pandemic and were willing to use them. They were also aware of the different measures put in place to prevent the further spread of the virus and were willing to follow them to ensure they received the service they were looking for. Most of the women followed the prevention measures and were able to get the services:

*“I did not experience any challenges, I just accepted that that was put in place, and I just had to follow it……... We just needed to follow all the rules they had put in place whether it meant wearing masks or washing hands, we just needed to follow that. And we really followed it. When we go to the hospital, we made sure we were wearing masks.” (Participant 020)*

#### Household expectations for utilization.

Most household members were highly receptive of FP and HIV services during the pandemic. Most participants were actually encouraged by their husbands to seek FP methods at the clinic to prevent pregnancies and also ensure ART continuity during the pandemic. Some also reported getting some support from other family members and friends.

*“He (husband) said I should not be afraid of COVID-19; I should still get the family planning; if I do not, we might have a child, as a result it may affect our child spacing. He said that a woman needs some time before giving birth to another child. That encouraged me to go to the hospital.” (Participant 001)*

*“Me and my husband just encourage each other, and we remind one another whenever it is time to say ‘tomorrow is your refill day, don’t forget’ and that is it.” (Participant 017)*

#### Financial accessibility.

Participants were asked about their finances during the pandemic. They were specifically asked about their jobs and business during the pandemic and whether or not it was affected by the pandemic.

Almost all of them reported that their finances were impacted negatively by the pandemic. Some reported losing their jobs as they were laid off; others reported some reduction in their hours of work, which reduced total earnings, and also loss of business due to fewer customers patronizing them.

*“During that time most companies had let some employees go. For a family like mine that depends on working for these employees, it was hard to find work because they were not working as well, this made providing food to our families hard.” (Participant 005)*

*“Ee, I cannot really explain that because we were doing businesses and it was still quite hard to do… At the time, people were scared, to say ‘if I go there, maybe I will contract the virus’. Some understood, and they were wearing the masks, but still, it was quite hard because the disease had really spread.” (Participant 011)*

Very few of the participants were able to pay for the services at a private facility. These few opted for private service providers after they failed to receive the service at the government facility within their area.

#### Challenges.

Although most participants were eventually able to access the services they were looking for, they faced different challenges. The most commonly reported challenges were: being sent away if they did not have a mask, being sent away if the maximum number of clients per day had been reached, and higher fares for the few who needed to take a minibus. Some also reported FP commodity stockouts on the day they visited the facility. Those who had challenges with transportation to the facility were those who accessed ART at another facility other than their closest one, which some women preferred to do for privacy reasons.

*“During COVID, I can say that transport was my main challenge. There were days I failed to come to the hospital in time because I did not have any transport money since it was hard to find work in order to earn money.” (Participant 028)*

Apart from fears of contracting the virus when they went to public places, some also had fears of being forced to take the COVID-19 vaccine if they went to the facility as they heard rumors that getting the vaccine was a prerequisite to being assisted.

## Discussion

This mixed methods analysis documented access to FP and HIV services among women during the COVID-19 pandemic and also explored challenges faced while accessing or trying to access the services. We found that overall, access to these services were sustained, and that women followed the preventive measures being implemented to reduce the spread of the virus. They, however, faced a number of different challenges and uncertainties while accessing the services. However, FP clinic had a specific number of clients to serve in a day to avoid overcrowding and most FP clients reported that they were sent away when the maximum number for the day was reached as access was first-come, first-serve basis.

Majority of women perceived FP and HIV as an important service, and most of them accessed them during the pandemic as they valued the need to prevent pregnancy and/or to be consistently taking ART. This finding also indicates the resilience of the health system during the pandemic as it implies the services were available. Such findings have also been observed in other studies evaluating FP [5,13,36] and HIV service access during the pandemic [37]. Our findings are, however, contrary to other studies where women perceived FP as less important since it does not have an immediate direct impact on health and illness [38]. In this study, the need to prevent pregnancy during the pandemic could also have been a result of the economic challenges and uncertainties brought by the pandemic itself as people may avoid life changing decisions during uncertain times.

Although women in our study had a positive perception about FP and HIV services access during the pandemic, almost all participants expressed fear of contracting COVID-19 either on their way to the clinic or at the clinic itself. This fear ultimately did not deter them from accessing the services, and they made sure they followed all preventive measures introduced by the government, including wearing of masks and observing social distance. Our results do, however, contrast with other studies that found that fear of contracting COVID-19 was one of reasons for not accessing services at the facility [10,39,40]. A qualitative study in Egypt reported that many participants were unable to access maternal health and FP services during COVID-19 due to fear of contracting the virus [10].

Some measures aimed at preventing the further spread of the virus also led to reduced household income. Our findings indicate that almost all women interviewed were negatively impacted by the COVID-19 pandemic either through loss of business, loss of job for them or their spouse, and/or reduction in hours worked. Limited finances can hinder access to health care [38,41]. In our study, however, the reduced monthly income did not significantly impact access to FP and HIV services as most women relied on free FP and HIV services at the government facility, which was within walking distance.

Apart from the self-initiative for our participants to use FP and HIV services, the high use of the services during the pandemic could also be attributed to the support they received. Most reported having support from their spouses and relatives to access the service. In Malawian settings, husbands/partners and other household members are crucial in influencing decisions and also mobilizing resources for health care access, including FP and HIV services. Particularly during the pandemic, their involvement could help in providing correct information about the pandemic and encourage their spouses/ family members to access the services.

The main challenge reported amongst FP clients was that they were sent away when the maximum number for the day was reached as access was first-come, first-serve, and the short-acting methods (condoms, pills, injectable) were given as a 3-month supply. The ART clinics did not have this challenge, as appointments are scheduled in advance for the next batch of ART medications. In addition, during the pandemic, the ART clinics started dispensing a 6-month supply (instead of a 3-month supply), which also helped to decongest the ART clinic [24]. Other reported challenges were lack of masks when it was a requirement, misinformation about COVID-19 and its associated vaccine, lack of transport for a few who needed money for transportation, and FP stockouts. These challenges also were reported in other studies, though in some, transportation was more pronounced due to travel bans implemented in their countries [10,42,43].

This study had some limitations. First, we only interviewed women in Area 25, a peri-urban area, so the results may not apply to all women, especially those from rural areas who cannot walk to their clinics. Conducting a similar study for rural women could be important to understand their experiences. The study also restricted interviews to the oldest woman per household who was the primary caretaker. This approach fails to capture the experiences of younger co-resident women, potentially limiting the generalizability of the findings to the broader female population in the study area. In addition, data was collected retrospectively and may be subjected to recall bias as we asked about their access to FP and HIV services during the pandemic period, which for Malawi ranged from April 2020 to May 2023. To minimize this bias, we tried to phrase our questions with a specific timeline and descriptions of events that occurred to ensure they restricted their responses to this time period and allow for a genuine recollection of events. All responses in this study were self-reported by the participants and not otherwise verified. We also acknowledge that these results may have been affected by social desirability bias as people tend to report what they perceive to be socially acceptable. We tried to minimize this by being neutral and non-judgmental during data collection and also ensuring confidentiality. There is also a potential selection bias inherent in the systematic sampling design, which selected every 10th household. If specific patterns exist within the layout of the study area, certain demographic groups may be under-covered or over-covered, thereby compromising the external validity and generalizability of the findings. Lastly, access to health care is impacted by individual, community, and facility factors. We did not interview heath facility staff, but tried to learn about facility-related factors, such as availability of staff and commodities at the facility, from the clients within their community.

These limitations, however, do not undermine the key implications that the study offers, including that FP and HIV service access was generally maintained in this peri-urban area during the COVID-19 pandemic. This finding could be because strict measures, such as a complete lockdown, were not implemented in Malawi, which afforded women the opportunity to move freely to the facility to access the services. They did still encounter challenges related to COVID-19 and its associated preventive measures while accessing or trying to access the services, particularly among those seeking FP services when compared to those seeking HIV services. Our findings emphasize the need to implement strategies that will minimize challenges to health care access during pandemics, to ensure gains in FP and HIV services are sustained. Future research could evaluate the long-term effectiveness of multi-month dispensing of ART and FP methods or self-care interventions (self-testing for HIV, self-injectable contraceptives, etc) to see if they could decrease barriers to accessing HIV and FP services, particularly during public health emergencies when services at health facilities may be more restricted. Finally, it would be important to perform additional qualitative inquiries into the lived experiences of those experienced unintended pregnancies during the pandemic and their long-term sequelae.

## Supporting information

S1 Table Precision indicators for Family Planning and HIV Service and Other related outcomes. (DOCX)
