# Supplementary material for: Assessing the impact of COVID-19 on access to family planning and HIV services among women in Lilongwe, Malawi
Source: PLOS Glob Public Health. 2026 Aug 11;6(8):e0007041. doi: 10.1371/journal.pgph.0007041 (PMC13460564; doi:10.1371/journal.pgph.0007041)
Supplement: Supplementary file 1 [file pgph.0007041.s001.docx]

| **Variable** | **Yes**  **Number (%)** | **Standard Error (SE)** | **Relative Standard Error (RSE)** |
| --- | --- | --- | --- |
| **Ever Use of FP** | 356 (89.0) | 0.015 | 1.76 |
| Were using FP when COVID started (April 2020) | 275 (77.2) | 0.222 | 2.88 |
| **Got pregnant during COVID-19 pandemic** | 194 (48.5) | 0.025 | 5.15 |
| Pregnancy was unintended | 65 (33.5) | 0.034 | 10.14 |
| **Wanted FP during COVID-19 pandemic** | 276 (69.0) | 0.023 | 3.36 |
| Tried to access FP (out of those who wanted FP) | 269 (97.6) | 0.009 | 0.97 |
| Cancelled/delayed FP visit due to COVID-19 | 51 (18.5) | 0.023 | 12.67 |
| COVID 19 affected choice of where they tried to  access FP (source) | 84 (30.4) | 0.028 | 9.12 |
| Obtained FP (out of those who tried) | 254 (94.4) | 0.014 | 1.48 |
| COVID-19 affected choice of FP method | 43 (16.0) | 0.022 | 14.00 |
| Received preferred method | 248 (92.2) | 0.016 | 1.78 |
| COVID-19 affected why they did not receive  preferred method | 17 (81.0) | 0.088 | 10.85 |
| **Eligible for HTC during COVID-19 pandemic** |  |  |  |
| Wanted HTC during COVID-19 pandemic | 302 (82.3) | 0.020 | 2.43 |
| Attempted to access HTC | 293 (97.0) | 0.010 | 1.01 |
| Accessed HTC | 290 (98.9) | 0.006 | 0.59 |
| COVID-19 restrictions affected access to HTC | 95 (31.5) | 0.026 | 8.49 |
| **Eligible for ART during COVID-19 pandemic** |  |  |  |
| Attempted to access ART | 33 (100) | - | - |
| Accessed ART | 33(100) | - | - |
| Had difficulty obtaining ART* | 2 (6.1) | 0.042 | 69.60 |
| Diagnosed with ART resistance* | 2 (6.1) | 0.042 | 69.60 |
| COVID-19 restrictions affected ART access* | 2 (6.1) | 0.042 | 69.60 |

S1 Table: Precision indicators for Family Planning and HIV Service and Other related outcomes

**The estimate should be interpreted with caution as the RSE >50, therefore not stable.*
